# Beneficial Effect of Synbiotic Supplementation on Hepatic Steatosis and Anthropometric Parameters, But Not on Gut Permeability in a Population with Nonalcoholic Steatohepatitis

**DOI:** 10.3390/nu8070397

**Published:** 2016-06-28

**Authors:** Silvia M. Ferolla, Cláudia A. Couto, Luciana Costa-Silva, Geyza N. A. Armiliato, Cristiano A. S. Pereira, Flaviano S. Martins, Maria de Lourdes A. Ferrari, Eduardo G. Vilela, Henrique O. G. Torres, Aloísio S. Cunha, Teresa C. A. Ferrari

**Affiliations:** 1 Departamento de ClínicaMédica, Faculdade de Medicina, Hospital das Clínicas, Universidade Federal de Minas Gerais, Belo Horizonte 30130-100, Brazil; clacouto@hotmail.com (C.A.C.); geyzaarmiliato@hotmail.com (G.N.A.A.); cristiano_asp@hotmail.com (C.A.S.P.); lferrari@medicina.ufmg.br (M.L.A.F.); egarciavilela985@gmail.com (E.G.V.); henrique.gamatorres@gmail.com (H.O.G.T.); aloisio@medicina.ufmg.br (A.S.C.); 2 Departamento de Anatomia e Imagem, Faculdade de Medicina, Universidade Federal de Minas Gerais, Belo Horizonte 30130-100, Brazil; lucianacosta@me.com; 3 Departamento de Microbiologia, Instituto de Ciências Biológicas, Universidade Federal de Minas Gerais, Belo Horizonte31270-901, Brazil; flaviano@icb.ufmg.br

**Keywords:** nonalcoholic fatty liver disease, nonalcoholic steatohepatitis, hepatic steatosis, probiotic, synbiotic, gut microbiota, intestinal permeability, lipopolysaccharide, obesity, weight loss

## Abstract

Nonalcoholic fatty liver disease is the most prevalent chronic liver disease in Western countries; it can progress to nonalcoholic steatohepatitis (NASH), cirrhosis and hepatocarcinoma. The importance of gut-liver-adipose tissue axis has become evident and treatments targeting gut microbiota may improve inflammatory and metabolic parameters in NASH patients. In a randomized, controlled clinical trial, involving 50 biopsy-proven NASH patients, we investigated the effects of synbiotic supplementation on metabolic parameters, hepatic steatosis, intestinal permeability, small intestinal bacterial overgrowth (SIBO) and lipopolysaccharide (LPS) serum levels. Patients were separated into two groups receiving *Lactobacillus reuteri* with guar gum and inulin for three months and healthy balanced nutritional counseling versus nutritional counseling alone. Before and after the intervention we assessed steatosis by magnetic resonance imaging, intestinal permeability by lactulose/mannitol urinary excretion and SIBO by glucose breath testing. NASH patients presented high gut permeability, but low prevalence of SIBO. After the intervention, only the synbiotic group presented a reduction in steatosis, lost weight, diminished BMI and waist circumference measurement. Synbiotic did not improve intestinal permeability or LPS levels. We concluded that synbiotic supplementation associated with nutritional counseling seems superior to nutritional counseling alone for NASH treatment as it attenuates steatosis and may help to achieve weight loss.

## 1. Introduction

Nonalcoholic fatty liver disease (NAFLD) is the most prevalent chronic liver disease in Western countries, and it is predicted that by 2030 this disorder will be the most common indication for liver transplantation worldwide [1]. NAFLD encompasses a spectrum of liver disorders characterized by hepatic steatosis that cannot be assigned to alcohol consumption. This condition ranges from simple steatosis to nonalcoholic steatohepatitis (NASH), which may present different grades of fibrosis and can progress to liver cirrhosis, and its related complications including hepatocellular carcinoma [2]. Recent evidence suggests that NAFLD is a multisystem disease that is not confined to liver-related morbidity and mortality, but also affects regulatory pathways and extra-hepatic organs particularly increasing the risk of type 2 diabetes mellitus (T2DM) and cardiovascular diseases [3]. Several clinical trials have been performed to identify a pharmacologic agent to treat NAFLD/NASH [4,5,6,7,8,9], but currently there is no approved specific drug for the treatment of this condition [10].

Although the complex pathogenesis of NAFLD is not fully elucidated, it is well known that this condition is strongly associated with insulin resistance (IR), visceral obesity and dyslipidemia [11]. The first hypothesis proposed to explain the pathogenesis of NAFLD is the “two-hit” theory. The “first hit” is characterized by lipid accumulation in the liver due to IR [12]; and the “second hit” is represented by lipid peroxidation, secretion of pro-inflammatory cytokines, and mitochondrial dysfunction determining the progression of the disease [12,13]. Currently, it is known that these mechanisms are not sufficient to explain all NAFLD pathogenesis; thus, the “multiple parallel hits hypothesis” has been attracted more attention. According to this conception, several parallel processes such as adipose tissue derived signals, gut barrier dysfunction, genetic factors, endoplasmic reticulum stress, and related signaling networks may act together causing the progression from steatosis to NASH [14].

It was previously demonstrated that small intestine bacterial overgrowth (SIBO) due to a jejunoileal bypass in obese surgical patients accelerates the progression of NAFLD because of the increased exposure of the liver to gut bacterial products [15]. More recently, evidence from experimental and humans studies supports the hypothesis that the gut microbiota may play a role in the pathogenesis of NASH by releasing lipopolysaccharide (LPS), increasing the production of ethanol, and activating inflammatory cytokines in the luminal epithelial cells as well as liver macrophages [16]. In this context, some clinical trials have demonstrated that modulation of the intestinal microbiota with probiotics (microorganisms that, when administered in adequate amounts, confer beneficial properties for the host) or prebiotics (non-digestible carbohydrates that affect the host beneficially by selectively stimulating proliferation and/or activity of populations of desirable bacteria in the colon) or synbiotic (formula containing probiotic plus prebiotic) supplements exerts beneficial effects in NAFLD progression [17,18,19,20,21,22,23].

The human gastrointestinal tract houses three dominating bacterial phyla: the gram-positive Firmicutes and Actinobacteria, and the gram-negative Bacteroidetes. Firmicutes is the largest bacterial phylum, comprising 200 genera, which includes *Lactobacillus* and *Clostridium*. *Bifidobacterium* is the major genus belonging to the phylum Actinobacteria. *Lactobacillus* is the most prevalent genus in the small bowel, whereas *Bifidobacterium* predominates in the large intestine [24]. Both are commonly used in the production of commercially available probiotic supplements.

In animal models, *L. reuteri* was associated with increased liver β-oxidation, reduction of the adipose and liver weights [25], and changes in the host immune system composition into a more anti-inflammatory profile, which may explain the decrease in body fat [26]. Inulin is a polysaccharide produced by various plants. It is categorized as a “non-digestible” oligosaccharide due to its resistance to hydrolysis by the human small gut digestive enzymes. Inulin is considered a prebiotic because it is fermented to short chain fatty acids (SCFAs) and lactate by bacteria from the colon [27], which stimulates the growth of beneficial bacteria [28]. Guar gum is a water-soluble, non-gelling fiber derived from the seeds of the drought tolerant plant *Cyamopsistetra gonoloba*, a member of Leguminosae family. It also has prebiotic properties as it increases the colonic contents of SCFAs, favoring the growth of *Lactobacillus* and *Bifidobacterium* [29].

To assess the clinical efficacy of *L. reuteri* with partially hydrolyzed guar gum and inulin in the treatment of NASH, we performed a randomized controlled clinical trial evaluating the grade of steatosis, presence of increased gut permeability and SIBO, and serum concentrations of LPS at baseline and at the completion of the synbiotic supplementation treatment in NASH patients.

## 2. Materials and Methods

### 2.1. Subjects

A controlled clinical trial was conduct on 50 patients with NASH attended at the Nonalcoholic Fatty Liver Disease Outpatient Clinic, Hospital das Clínicas, Universidade Federal de Minas Gerais, Belo Horizonte, Brazil, during a one-year period (2014–2015). This institution is a referral center of the Brazilian public health system. The inclusion criteria comprised: (1) diagnosis of NASH confirmed by liver biopsy (performed according to clinical judgment); and (2) exclusion of other causes of liver disease.

The patients were randomly assigned into two groups: those who received the synbiotic (*n* = 27) or those who did not received it and formed the control group (*n* = 23). We planned to form two similar groups in relation to the severity of the hepatic disorder, and the metabolic, clinical and anthropometric parameters to avoid confounding variables.

For the inclusion in the study, it was required that all patients have undergone liver biopsy previously. In order to confirm the diagnosis of NASH, an experienced pathologist who was blinded to the clinical data reviewed all but one slide and scored steatosis, lobular inflammation, ballooning injury and fibrosis according to the NASH Clinical Research Network (CRN) system: steatosis affecting >5%–33% (1 point), 34%–66% (2 points), and >66% of the hepatocytes (3 points); lobular inflammation in less than foci/×200 (1 point), 2–4 foci/×200 (2 points), and >4 foci/×200 (3 points); and ballooning of scarce hepatocytes (1 point), and several cells exhibiting prominent ballooning (2 points). The activity score of disease (NAS) was obtained adding the points: <3, NASH exclusion; ≥4, probable NASH; and ≥5: definitive diagnosis of NASH [30]. Patients with NAS <3 were not included in this survey.

As recommended by the American Gastroenterological Association, in all cases, other causes of liver disease were ruled out (namely: alcohol intake >20 g/day for males and >10 g/day for females, chronic B or C hepatitis virus infections, auto-immune hepatic disorders, Wilson disease, hemochromatosis and alpha-1-antitripsin deficiency) as well as other causes of hepatic steatosis or liver damage (use of steatogenic medications within the past six months, exposure to hepatotoxins and history of bariatric surgery) [11]. Alcohol use was addressed on at least three different occasions, by two doctors and by a dietician during a nutritional interview.

Exclusion criteria comprised: (1) withdrawal from the study; (2) presence of contraindication to magnetic resonance imaging (MRI) examination; and (3) evidence of decompensated liver disease, such as history or presence of ascites, bleeding varices, or hepatic encephalopathy.

All participants underwent anthropometric and body composition evaluations, serum biochemistry and hormones measurement, abdominal MRI, urinary excretion of lactulose and mannitol test, glucose hydrogen breath test and determination of serum levels of LPS at baseline and after treatment with synbiotic supplementation (synbiotic group) or at baseline and three months later (control group). The study was approved by the Ethical Committee of the Universidade Federal de Minas Gerais (CAAE—01699512.1.0000.5149) and all the patients signed the inform consent form.

### 2.2. Laboratory Evaluation

The laboratory assessment was performed after an overnight fast and included liver biochemistry (alanine aminotransferase (ALT), aspartate aminotransferase (AST), bilirubin, gamma-glutamiltransferase (GGT), alkaline phosphatase (AP) and albumin) and metabolic parameters (lipid profile, uric acid and fasting serum insulin and glucose). All tests were performed at the Central Laboratory of Hospital das Clínicas, Universidade Federal de Minas Gerais. Plasma glucose levels, bilirubin, albumin, triglycerides, and total cholesterol and fractions were quantified by colorimetric/dry chemistry assay (VITROS^®^ 5600 Integrated System, Hong Kong, China). Insulin was measured by chemiluminescence immunoassay (Architect I1000SR^®^, Wiesbaden, Germany) and IR was defined by HOMA values >3 [31]. ALT, AST, AP and GGT were measured by enzymatic kinetic/dry chemistry assay (VITROS^®^ 5600 Integrated System, Hong Kong, China). Platelet count was determined by an automated hematology analyzer (Sysmex XN10, Chuo-ku, Japan).

NAFLD fibrosis score [32] and all the other laboratory evaluations were determined at baseline and after the treatment (synbiotic group) or three months after the first tests (control group).

### 2.3. Nutritional Assessment

The anthropometric data included height (m), weight (kg), body mass index (BMI; kg/m^2^) and waist circumference (cm). Overweight was defined as BMI >25 and <30 kg/m^2^, and obesity as BMI >30 kg/m^2^ or waist circumference ≥80 cm (women) or ≥90 cm (men) [33]. Based on the measurement of waist circumference and the presence of metabolic disorders, the patients were classified as having metabolic syndrome (MS) according to the criteria defined by the International Diabetes Federation [34].

Electrical bioimpedance was used to determine body composition (Biodynamics model 450, version 5.1, Seattle, WA, USA). Fat-free mass (expressed in kg and percentage), fat mass (expressed in kg and percentage) and the basal energy expenditure (expressed in kcal) were recorded. In order to obtain adequate test accuracy, all patients were informed about the correct preparation: four-hour fasting, no performing vigorous physical activities in the 48 h before the exam, and no alcohol consumption for at least 48 h before the exam. The amount of body fat was classified as high when it was above 25% and 35% for men and women, respectively [35,36].

### 2.4. Magnetic Resonance Imaging

Considering the impossibility of performing liver biopsy before and after the intervention because of the risks of the procedure, we assessed at baseline and at the end of the study, using MRI techniques, liver steatosis by measuring the hepatic proton density fat fraction (PDFF) [37,38,39,40,41,42,43,44,45], and liver fibrosis using elastography [46]. The subjects were asked to fast at least four hours and were examined in the supine position with a 1.5 Tesla eight-channel torso phased-array coil (SignaHDxt, GE Medical Systems, Milwaukee, WI, USA) centered over the liver. A dieletric pad was placed between the coil and the body wall. To estimate MRI PDFF, unenhanced axial images were obtained by using a low-flip-angle, two-dimensional spoiled gradient-recalled-echo sequence with all array coil elements as described previously [43,47,48]. By using a research software algorithm that runs on AW 4.6 workstation, MRI PDFF maps were generated pixel by pixel from the source images. Trained image analysts who were blinded to clinical and histological data reviewed the MR images and manually placed free-hand regions of interest (ROIs) in three images: the first one at the level of the portahepatis and the other two below and above this level, avoiding major vessels, liver edges, and artifacts on the MRI PDFF maps in each subject. The PDFF in each of the three ROIs was recorded, and the PDFF value across the entire liver was reported as the mean of the PDFF values of all the three ROIs.

The classification of the grade of steatosis was described according to the method previously published by Tang et al. [45], which defines MRI PDFF threshold from the NASH CRN study [30] considering the following PDFF thresholds: 6.4% for differentiating grade 0 from steatosis grade ≥1; 17.4% for differentiating steatosis grade ≤1 from grade ≥2; and 22.1% for differentiating steatosis grade ≤2 from grade 3 [45]. For the statistical analysis we grouped the grade 0 with grade 1 and grade 2 with grade 3.

Relaxometry methods were performed to calculate T2* by fitting the decay models to the average signal intensity at various echo times (ETs). These values were expressed as relaxation rates R2* (1/T2*) and a T2 parametric map was automatically obtained. This sequence was obtained to investigate the presence of iron overload, as this situation may obscure fat quantification.

We used MR elastography to determined liver fibrosis. This method was chosen because it evaluates larger liver volumes and is unaffected by obesity [46]. MR elastography was performed according to the established method as previously published [49]. Briefly, MR elastography was performed with a 1.5-T whole-body imager (Signa, GE Medical System, Milwaukee, WI, USA) by using a transmit-receiver coil. Continuous longitudinal mechanical waves at 60 Hz were generated using an acoustic pressure waves-transmitted driver device on the anterior chest wall. A two-dimensional gradient-echo MR elastography sequence was performed to acquire axial wave images with the following parameters: repetition time ms/echo time ms 50/23; continuous sinusoidal vibration, 60 Hz; field of view, 32–42 cm; matrix size, 256 × 64; flip angle, 30°; section thickness, 10 mm; four evenly spaced phase offsets; and four pairs of 60-Hz trapezoidal motion-encoding gradients with zero and first moment nulling along the through-plane direction. All processing steps were applied automatically to yield quantitative images of tissue shear stiffness in kiloPascal (kPa). An abdominal radiologist with 15 years of experience performed interpretation of the MR elastographic images. The liver stiffness was classified considering a cut-off value of 2.93 kPa for discriminating any grade of hepatic fibrosis from normal liver tissue [50].

### 2.5. Intestinal Permeability Test

After a 10-h fast, the residual urine was discharged and immediately after this procedure, the patient drank an isosmolar solution (120 mL) containing 6.0 g of lactulose and 3.0 g of mannitol. Over a period of five hours, the urine was collected in a sealed bottle. After that, 2.5 mL of urine was stored in a second smaller bottle and 0.6 mg thimerosal was added to avoid bacterial growth. The samples were stored in liquid nitrogen. Urinary lactulose and mannitol were analyzed by high performance liquid chromatography (HPLC) using a Shimadzu^®^ system (Kyoto, Japan). Briefly, after filtering the urine through a micropore filter (0.22 μL, Millex, São Paulo, Brazil), it was passed through an ion-exchange resin (Mixed-bed resin TMD-8, Sigma, St. Louis, MO, USA), and then 50 μL was injected into the chromatograph with an auto-injector. MilliQ water was used as mobile phase at a predetermined flow rate of 0.6 mL/min. A Supelcogel 33H^®^ pre-column (St. Louis, MO, USA) and a Rezek RHM monosaccharide H+ (8%)^®^ column (Torrence, CA, USA) were used for separating the substances sequentially. The waves generated by lactulose and mannitol were captured and analyzed by the workstation software [51]. The results are reported as the percentage of urinary excretion of each probe in relation to the amount ingested; and the final result of the test, as the ratio between the excreted percentages of lactulose and mannitol. This ratio is a more accurate indicator of permeation as it minimizes the influences of the factors that can affect the absorption of the probes [52]. The reference value for urinary excretion of lactulose was <0.195; for urinary excretion of mannitol, >4.08 and for the ratio of lactulose/mannitol, <0.0157. These reference values were determined in a healthy population in a previous study conducted by the same authors of the present study [53].

### 2.6. Glucose Hydrogen Breath Test

Glucose hydrogen breath test (H2BT) was performed using a portable breath hydrogen monitor (Gastrolyzer; Bedfont Scientific Ltd., Maidstone, UK) at the baseline and at the end of the study. All patients were instructed not to eat fermentation food in the 24 h before the test and not to use antibiotics in the 14 days preceding the test. After an overnight fast, one sample of hydrogen exhalations in breath was taken as basal breath hydrogen level. Then, the subjects drank 50 g of glucose dissolved in 200 mL of water within 5 min. Thereafter, breath hydrogen exhalation was determined every 15 min for the next 60 min; and from the next hour, every 30 min for a total time of 2 h for testing. A rise in breath hydrogen of 15 ppm within the first 80 min after glucose ingestion was considered indication of SIBO [54].

### 2.7. Measurement of Serum LPS

Peripheral blood of all patients was collected and serum samples were stored at −20 °C in bottles free from pyrogens. Glassware used for the determination of LPS was heated at 250 °C for 120 min to remove non-specific inhibitors of endotoxin. LPS plasma levels were then determined using a commercial kit (Limulus QCL-1000, Amebocyte Lysate, Lonza, Walkersville, MD, USA) according to the instructions of the manufacturer.

### 2.8. Synbiotic Supplementation and Dietary Intervention

In order to select the commercial synbiotic product, we conducted a microbiological evaluation of at least three different manufacturers to guarantee that the product had the recommended number of colony forming units (CFU). The synbiotic used in the present study was considered appropriate in this requirement.

The study group received 5 g of the synbiotic (Fiber Mais Flora^®^, Nestlé Health Science, Osthofen, Germany), which consisted of 4 g of dietary fiber (partially hydrolyzed guar gum and inulin) and 1 × 10^8^ CFU of *L. reuteri*, twice daily during three months. All patients were advised about the way to conserve and intake the synbiotic supplementation. Nutritional appointments were scheduled every month to provide the synbiotic, verify adherence to the diet and determine the anthropometric parameters. Control individuals were also evaluated monthly in the same way as the study group.

For dietary intake investigation, we applied three non-consecutive 24-h dietary recalls [55], one in each appointment, in order to determine adherence to treatment and to adapt the diet of the patient to the recommended guidelines when necessary. The 24-h dietary recall is a suitable tool to assess food and beverage intake within the previous 24 h. It is easy to apply, is inexpensive, and does not depend on the respondent’s literacy.

Analysis of food intake consisted of the calculation of total energy consumed. The estimated energy intake from the first 24-h dietary recall of each patient was obtained from the Brazilian Food Composition Table [56]. Based on the average energy intake we established a food plan that provided 1500 kcal for women and 1800 kcal for men, which is in agreement with the recommended reduction of 500 to 1000 kcal/day in relation to the current diet of the NAFLD patients [57]. All patients also received general nutritional instructions to achieve healthy balanced diet based on The Dietary Guide for Brazilians, which consists of the guidelines proposed by the Brazilian Ministry of Health according to the recommendations of the World Health Organization [58].

At the three monthly visits, the subjects’ doubts were answered and patients were encouraged to remain adherent to a healthy balanced nutritional counseling and synbiotic supplementation. After the three-month intervention, we calculated the median value of energy intake, which was compared between the groups.

The patients were also encouraged to keep their usual physical activities during the study. According to the American College of Sports Medicine and the American Heart Association criteria [59], they were classified as physical activity practicing when they practiced physical activity for 30 min at least five times a week.

### 2.9. Statistical Analysis

Statistical analyses were performed using the SPSS software, version 18 (SPSS Inc., Chicago, IL, USA). The data are presented as frequencies, proportions, means ± standard deviations (SD), and medians and range. The Shapiro-Wilk test was used to determine whether continuous variables were normally distributed. Continuous variables were compared between intervention and control groups using the *t*-test (normal distribution) or the Mann-Whitney U test (asymmetrical distribution), and proportions were compared using the chi-square test or the Fisher’s exact test, where appropriate. The paired *t*-test or the Wilcoxon test was used to compare data between the first and second evaluations, for normal or asymmetrical distributions, respectively. To compare the frequencies of paired variables, we used the McNemar test. For all tests, *p*-values < 0.05 were considered statistically significant.

## 3. Results

### 3.1. Characteristics of the Patients

Of the 50 patients included in the study, the median age was 57.3 years (range 25–74 years) and there was no difference in the median age between the groups (*p* = 0.365). Thirty-eight patients were female (76%) and similarly there was no difference in the proportion of males and females between the groups (*p* = 0.325). Table 1 presents the histological features of the total population before the inclusion. The grades of steatosis, lobular inflammation, ballooning and fibrosis identified in the liver biopsies performed before inclusion in the study were similar between the groups.

Although the median values of PDFF measured by MRI were higher in the study group at baseline, when we grouped the patients according to the grade of steatosis, there was no difference between the study and control groups concerning the proportion of individuals classified as having mild or moderate/severe steatosis (Table 2). Regardless the chosen method to estimate liver fibrosis (MRI elastography or the NAFLD score), the proportion of patients having liver fibrosis was similar between the groups (Table 2). Liver biochemical parameters were similar in both groups.

Most patients were obese, hypertensive, dyslipidemic, and sedentary; presented alterations in the glucose metabolism; and were classified as having the MS. The comparison between the baseline clinical data of the two groups regarding the metabolic characteristics and hepatic biochemical parameters showed no differences (Table 3), reinforcing the homogeneity of the groups at the time of study enrollment.

At baseline, the patients demonstrated low frequency of SIBO (2% in the total population); however, 51.1% of them presented increased intestinal permeability. The intestinal parameters are presented in Table 4. There was no difference regarding these data between the groups.

All the patients completed the three months of the study with good adherence to therapy as documented by themselves in the monthly nutritional appointments. No adverse effects were reported.

### 3.2. Main Outcomes

#### 3.2.1. The Effect of Synbiotic on Fatty Liver Evaluated by MRI

**Figure 1:**
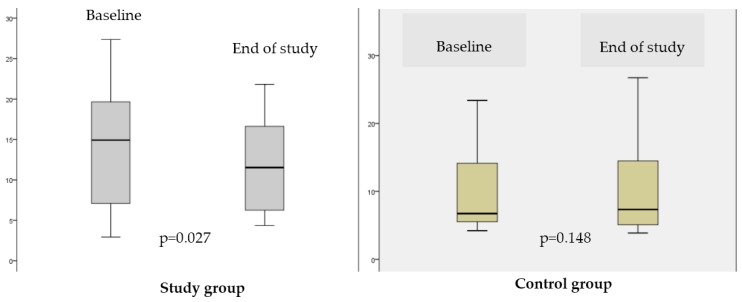
Comparison of the values of the hepatic proton density fat fraction (PDFF) using magnetic resonance image (MRI) in the study group and in the control group after intervention.

The reduction in hepatic steatosis after three months of supplementation with the synbiotic was the main result of the study. The median value of the MRI-PDFF decreased from 14.9 (range, 2.9–27.4) to 11.5 (range, 4.4–21.8), which represents an improvement in the hepatic steatosis (*p* = 0.027). In contrast, in the control group, the median values of the MRI-PDFF did not change: 6.4 (range, 3.9–23.4) at enrollment and 7.3 (range, 3.9–31.1) (*p* = 0.148) and end of the study (Figure 1).

In line with this result, 40.7% of the patients in the study group were classified as having moderate/severe steatosis (grades 2–3) in MRI at baseline; and, after the synbiotic supplementation, this proportion fell to 18.5% (*p* = 0.031). Therefore, the proportion of patients initially with mild steatosis (grades 0–1) increased from 59.2% to 81.5% (*p* = 0.031). In the control group, we did not observe improvement in the grade of steatosis (*p* = 1.00).

The synbiotic supplementation did not affect liver fibrosis. The proportion of cases classified by the NAFLD score as no fibrosis, indeterminate or significant fibrosis were similar between the groups before (*p* = 0.552) and after the intervention (*p* = 0.223). Similarly, in the MRI elastography evaluation there was no difference in the proportion of patients classified as having normal liver tissue (before: 29.2%; after: 41.2%) or any grade of fibrosis (before: 70.8%; after: 58.3%) (*p* = 0.252) at the beginning and the end of the follow up.

#### 3.2.2. The Effect of the Synbiotic on Metabolic Parameters and Hepatic Biochemistry

After the three-month synbiotic supplementation, the study group also presented a reduction of the anthropometric parameters. These patients lost 1.5% of their initial body weight, and BMI and waist circumference measure decreased by 1.2% and 1.8%, respectively. These results were not verified in the control group in which the anthropometric parameters remained fairly stable (Table 5). There was no difference in the median energy consumption throughout the study between the two groups: 1609.6 kcal (range, 1075.4–2006.9 kcal) in the study group and 1739.9 kcal (range, 1024.3–2208.8 kcal) (*p* = 0.330) and in the control group. Both groups were similar (*p* = 0.073) concerning the practice of physical activities throughout the study period.

Regarding biochemistry, the levels of uric acid decreased in the study group whereas very low-density lipoprotein cholesterol (VLDL-c) and triglycerides serum concentrations increased in the control group. Concerning liver biochemistry and other metabolic parameters, no changes were observed in both groups during the study (Table 5).

#### 3.2.3. The Effects of the Synbiotic on Intestinal Parameters: SIBO, LPS Concentration and Intestinal Permeability

The prevalence of SIBO was low in the studied population. H2BT was abnormal only in one patient of each group at baseline. After the intervention, the frequency of SIBO remained low without any significant difference when compared to the baseline in the study group (*p* = 0.125) as in the control group (*p* = 0.500).

Concerning the serum levels of LPS, at the end of the intervention, we observed significant increase in both groups. Compared to the baseline values, in treated patients, the mean serum LPS levels rose by 0.41 EU/mL (*p* = 0.000) and in the control group by 0.49 EU/mL (*p* = 0.004).

From the total population, most individuals (51.1%) presented high intestinal permeability at baseline. There was no difference in the percentage of lactulose and mannitol excretion or in the lactulose/mannitol excretion ratio in the intervention group (*p* = 0.492, *p* = 0.459, and *p* = 0.737, respectively) as well as in the control group (*p* = 0.248, *p* = 0.950, and *p* = 0.374, respectively).

## 4. Discussion

Our study showed that treatment with 10^8^ CFU of *L. reuteri* plus 4 g of partially hydrolyzed guar gum and inulin (twice a day) for three months reduced the grade of hepatic steatosis and improved some of the metabolic parameters associated with NASH, such as body weight, BMI, waist circumference and uric acid serum levels. The improvement on steatosis occurred despite no effects of the synbiotic supplementation on SIBO and parameters of gut permeability (urinary lactulose/mannitol excretion and serum LPS levels).

It is well established the close relationship between obesity and liver diseases [60]. Increased waist circumference indicates abdominal fat accumulation, which is associated with visceral adiposity, cardiometabolic risk factors, hepatic steatosis [61] and liver necroinflammatory activity [62]. Increased BMI is an independent predictor of development of severe fibrosis and hepatocarcinoma [63,64]. According to the current guidelines recommendations, lifestyle modifications focused on weight loss is the first line treatment of NAFLD [11]. In our study, after supplementation with the synbiotic, the patients lost 1.5% of the initial body weight, and presented a reduction of 1.2% in BMI and of 1.8% in the waist circumference measure. These results were associated with a reduction of 3.4 points in the median values of PDFF measured by MRI, suggesting decrease in liver fat accumulation.

Both the study and control groups were advised to follow the same healthy balanced nutritional counseling, were followed by a dietitian during the same time interval and showed similar median energy intake along the three months of the follow-up. Thus, we consider it reasonable to believe that the synbiotic may have helped to improve the anthropometric parameters in the study group. *Lactobacillus* supplementation has previously been related to weight loss in a multicenter, double-blind, randomized, placebo-controlled intervention trial involving adult population with obese tendencies. According to the results of that study, the use of *L. gasseri* for three months led to an average reduction of 1.4% in the body weight, 1.5% in BMI and 1.8% in waist measure [65]. These findings are very similar to those found in our study. The lack of investigation of the liver parameters in that study precludes comparisons of these parameters.

The mechanism by which probiotic supplementation could influence the loss of weight has not been fully elucidated. However, some studies demonstrated that intestinal microbes have an important role in body weight regulation by influencing energy metabolism [66,67]. Microbiota can increase energy harvesting from the diet and enhance energy storage contributing to the development of obesity. Obese subjects show less diversity of gut bacteria, and different expression of both bacterial genes and metabolic pathways in comparison to non-obese individuals [66,68]. It was demonstrated the relative lower proportion of Bacteroidetes and higher proportion of Firmicutes in the gut of obese individuals compared with lean people; furthermore, when obese subjects experiment weight loss, there is an increase in the proportion of Bacteroidetes [66]. In obese patients, this shift in the relative abundance of phyla is associated with elevation in the ability for harvesting energy from indigestible polysaccharides present in the diet, which are normally broken by glycoside hydrolases and polysaccharide lyases that are enzymes absent in humans [68,69]. The intestinal bacteria are able to convert these polysaccharides into monosaccharides and short-chain fatty acids in the colon leading to triglyceride synthesis in the liver [70]. A NASH patient seems to have also a lower proportion of Bacteroidestes/Prevotella in the stool when compared to the individuals with simple steatosis or healthy controls (living liver donors), independently of BMI and dietary fat intake [71]. Not only supplementation with probiotics is capable of modulating the gut microbiota, but also the prebiotic administration. It was recently demonstrated that supplementation with partially hydrolyzed guar gum stimulates *Bifidobacterium* and butyrate-producing bacteria in the human large intestine, which confers healthy benefits to host [72]. Based on the exposed data, we speculate that the modulation of microbiota by synbiotic treatment may hamper the development of other bacteria phyla that are more efficient in harvest energy from the diet; however, our study did not provide evidence to support this hypothesis as the investigation of the composition of the gut bacterial communities of our NASH patients was beyond its scope.

Based on evidence from experimental models, probiotics may inhibit lipid absorption contributing to the loss of weight. Sato el al. [73] observed a regulatory effect of *L. gasseri* on the size of adipocytes in rats. Furthermore, Hamad et al. [74] studied the lipid content of the lymph in rats with permanent cannulation of the thoracic duct and demonstrated that the animals fed a diet containing fermented skim milk with *L. gasseri* presented a lower rate of maximum lymphatic lipid absorption than the rats fed a diet containing non-fermented skim milk. The reduction in lipid absorption was also supported by the observation of increased fatty acid excretion in the feces. Indeed, probiotic bacteria seem to increase liver mitochondrial β-oxidation of fat determining both loss of weight and reduction in hepatic steatosis. This hypothesis is supported by the experimental study from Fak and Backhed [25]. They demonstrated that specific strains of *L. reuteri* were associated with minor weight gain, reduction in adiposity and liver weights, and increased expression of carnitine palmitoyltransferase 1a (Cpt1a) indicating increased β-oxidation in the mice fed a high-fat diet [25]. The possible mechanism involved in these benefits refers to the influence of probiotic bacterium on liver lipid metabolism and whole-body adiposity via increased expression of Cpt1a. It is important to consider that the same species of probiotic bacterium, may present different strains, which may cause different effects on adiposity, insulin sensitivity and liver lipid metabolism, illustrating the complexity of host-bacterial cross-talk and the importance of investigating specific bacterial strains.

The supplement used in our study contained in addition to the probiotic bacterium, prebiotic soluble fibers (4 g of partially hydrolyzed guar gum and inulin) administered twice/daily. Therefore, it is not possible to determine which component led to the improvement of the anthropometric parameters and liver steatosis. Dietary fibers are non-digestible carbohydrate, which are fermented by colonic bacteria producing SCFA that stimulates the secretion of glucagon-like peptide-1 (GLP-1) and regulate other appetite hormones [75]. In this context, dietary fibers could be linked to the reduction of energy intake; however, in our study we did not find any differences regarding energy intake between the study and control groups. Guar gum supplementation was not associated with weight loss in human subjects [76,77] and data about the effects of inulin in weight loss are conflicting. Studies involving children [78] and adults [79] did not confirm any positive effect of inulin supplementation on weight loss. Otherwise, evidence from experimental studies demonstrated that guar gum could reduce hepatic steatosis [80]. Similar findings were described in humans with glucose intolerance that received inulin supplementation and presented decrease in intrahepatocelullar lipid regardless of weight loss [81]. Thus, it is possible that prebiotic supplementation in our study has also contributed to reducing hepatic steatosis.

The other relevant finding of our study was the reduction in the serum uric acid levels after the synbiotic supplementation. Observational studies demonstrated that hyperuricemia is a risk factor for NAFLD among eastern Asian populations independently of the presence of the components of MS [82]. This association was late confirmed in a large population cohort from the United States [83]. The underlying mechanism of this association is the evidence that IR can reduce urinary excretion of uric acid and increase its serum levels [84]. In our study, the reduction of serum levels of uric acid was associated with improvement on the grade of steatosis, but was not accompanied by alterations of the insulin levels or HOMA-IR. In a recent systematic review and meta-analysis of randomized controlled trials, it was demonstrated that probiotic consumption, compared with placebo, significantly reduced fasting plasma insulin and HOMA-IR [85].

Although we have found a reduction in hepatic steatosis after the treatment, it was not reflected in improvement of the liver biochemistry and platelet count, since AST, ALT, AP, GGT, bilirubin, albumin and platelet number did not change compared to the baseline values. The effects of probiotic/synbiotic on liver biochemistry are conflicting. In the studies by Wong et al. [23] and Malaguarnera et al. [21], the reduction of steatosis was accompanied by decrease of the AST concentrations, but not of the other liver enzymes. Other studies [17,18] demonstrated reduction in AST, ALT and GGT concentrations, but the authors did not investigate the effects on liver fat. Vajro et al. [22] and Loguercio et al. [19] identified reduction only in ALT concentrations. However, it is important to highlight that liver enzymes are usually normal or minimally increased in NAFLD patients [86]. In this context, one of most important strengths of our study was the use of MRI to evaluate liver steatosis. The chosen method has a satisfactory accuracy for classifying the histological steatosis grades in the NAFLD population with a sensibility of 86%, 64% and 71%, and a specificity of 83%, 96% and 92% to detect steatosis grades 1, 2 and 3, respectively [45]. In our investigation, the intensity of the steatosis measured by MRI PDFF was higher in the study group in relation to the controls at baseline, which makes the reduction of this parameter at the end of the follow-up even more significant. As previously demonstrated, we observed a high prevalence (51.1%) of increased intestinal permeability in our NASH population [87,88]. Increased intestinal permeability may be related to the pathogenesis of NASH since it could expose the liver to LPS an active component of endotoxin from the gut bacteria. LPS in turn is involved in the activation of toll-like receptor 4 (TLR4) in the Kupffer cells stimulating essential inflammatory cascade [69], which results in prolonged liver inflammation, liver damage [89], and impairment of insulin signaling by diminishing phosphorylation of the insulin receptor [68]. IR increases lipid oxidation in the adipose tissue and, therefore, enhances the influx of nonesterified fatty acid to the liver [90]. Hyperinsulinemia increases the levels of sterol regulator element-binding protein-1c (SREBP-1c), which up-regulates lipogenic gene expression, increases lipogenesis and accelerates hepatic fat accumulation [91].

The fact that the increased intestinal permeability was not accompanied by SIBO, contradicting the findings of other studies [87,92,93,94,95], which demonstrated a large percentage of positive H2BTs in patients with NAFLD, is noteworthy. On the other hand, the high prevalence of SIBO in NAFLD individuals has been questioned by several investigators [22,96]. Similar to our results, Vajro et al. reported a very low frequency of SIBO in a NAFLD children population [22]. In our study, methodological aspects may have affected the ability of detecting SIBO, as the glucose H2BT may be false negative in the presence of SIBO due to bacteria that produce methane instead of hydrogen. In this situation, a breath test measuring methane exhalation, which was not performed in our investigation, could have detected bacteria overgrowth. However, the fact that our patients were mostly asymptomatic from a gastrointestinal viewpoint argues against the existence of SIBO.

After synbiotic supplementation, the prevalence of SIBO did not demonstrate significant changes as well as the intestinal permeability. Unexpectedly, we verified that the LPS concentrations increased in both group (rose by 59% in the synbiotic group and 77% in the control group). Contrary to our findings, Malaguarnera et al. demonstrated that the mean serum LPS levels fell by 33% and by 13% in the synbiotic and placebo groups, respectively [21]. We are not able to explain our results concerning LPS analysis, but it is not possible to completely rule out the possibility of technical limitations of method itself. Unfortunately, we did not measured LPS levels in healthy subjects, which would have enabled the characterization of normal/high LPS levels. It is reasonable to assume that synbiotic was not responsible for the increased in the levels of LPS, since this alteration was seem in both groups.

Taken together, our findings lead us to believe that the synbiotic should not have affected the gut microbiota and intestinal permeability, although it may have driven the reduction of the grade of steatosis in the NASH patients by another mechanism. As stated by several authors, synbiotic supplementation may have beneficial effects via immune-mediated mechanisms [97,98,99,100,101,102] that could improve the subclinical pro-inflammatory signaling cascade encountered in obesity [103] and reduce liver fat without changing the existing gastrointestinal microbiota. This hypothesis is supported by a beautiful experimental study, which demonstrated in a Swiss mouse model of obesity that *L. reuteri* prevented weight gain and these protective effects were irrespective of the baseline diet. Abdominal and subcutaneous fat accumulations were significantly reduced in the mice feeding purified *L. reuteri* in combination with either control or Western diet. It is noteworthy that *L. reuteri* acted without changing the gastrointestinal microbiota composition or level of calorie consumption [26]. Although probiotics may not restructure resident microbiota communities, a pre-existing diverse microbial composition seems to be required for optimal slenderizing effects as illustrated by the fact that mice raised under germ-free conditions, and then fed *L. reuteri* under general housing conditions, fail to benefit from taking the probiotic organisms. The slenderizing microbial mechanism should involve bacteria-triggered changes in the host immune system. Evidence suggests that this effect is particularly dependent on CD4+ T cells and on the anti-inflammatory interleukin (IL)-10, as IL-10 deficient animals were resistant to *L. reuteri* induced effects. Adoptive transfer of *L. reuteri* from IL-10-competent animals induced anti-inflammatory Foxp3+ Treg cells that decreased body fat in naive recipient animals [26].

As mentioned above, one limitation of our study was the lack of liver biopsy after the treatment; however, for ethical reasons, we did not repeat this procedure after the use of the synbiotic to evaluate hepatic histology. Another limitation is the fact that we did not evaluate the gut microbiome, which could have indicated the effects of the synbiotic consumption on gut microflora contributing to the understanding of its suggested mechanism of action. Furthermore, as stated, we did not measure LPS levels in healthy individuals, which could help in the interpretation of our results. Finally, we did not investigate other lifestyle factors with the potential to exert influence on our results. As stated by others, future large-scale intervention trials investigating the potential benefit of probiotics/synbiotics should include a combination of gastrointestinal function tests, including lactulose H2BT and methane breath test, intestinal permeability evaluation, and the study of the feces composition [22].

The most important strengths of the current study were the randomization design, the inclusion of only patients with NASH diagnosed by liver biopsy, and the evaluation of the investigated parameters especially intestinal permeability, SIBO and LPS before and after synbiotic treatment. All of these strengths are relevant in comparison with the few other clinical trials in which the effects of probiotics alone or in combination with prebiotics for treating NAFLD/NASH were evaluated [17,18,19,20,21,22,23,104,105,106].

## 5. Conclusions

In conclusion, this randomized controlled trial demonstrated some evidence that a three-month synbiotic supplementation in addition to lifestyle modification is superior to lifestyle modification alone for the treatment of NASH, as the intervention group presented attenuation of steatosis and reduction of body weight, BMI and waist circumference. This improvement occurred despite the lack of effects on gut permeability and SIBO. Whether these effects will be sustained with longer treatment duration remains to be determined.

## Figures and Tables

**Table 1:** Histological features in liver biopsies of the patients with nonalcoholic steatohepatitis before inclusion.

Variable	Total (*n* = 49)	Study Group (*n* = 26)	Control Group (*n* = 23)	*p* Value
**Steatosis**				0.321 ^¶^
5%–33%	12 (24.5%)	4 (15.4%)	8 (34.78%)	
34%–66%	27 (55.1%)	17 (65.4%)	10 (43.5%)	
>66%	10 (20.4%)	5 (19.2%)	5 (21.7%)	
**Lobular Inflammation**				0.08 ^¶^
<2 foci ×200	31 (63.3%)	17 (65.4%)	14 (60.9%)	
2–4 foci ×200	14 (28.6%)	5 (21.7%)	9 (34.6%)	
>4 foci ×200	4 (8.2%)	4 (17.4%)	0 (0.0%)	
**Ballooning**				0.166 ^¶^
Few balloon cells	24 (49.0%)	16 (61.5%)	8 (34.8%)	
Prominent balloon cells	25 (51.0%)	10 (38.5%)	15 (65.2%)	
**Fibrosis Stage**				0.502 ^¶^
F0	22 (44.9%)	12 (46.2%)	10 (43.5%)	
F1	16 (32.7%)	7 (26.9%)	9 (39.1%)	
F2	3 (6.1%)	3 (11.5%)	0 (0.0%)	
F3	5 (10.2%)	3 (11.5%)	2 (8.7%)	
F4	3 (6.1%)	1 (3.8%)	2 (8.7%)	
**NAS Score**				**0.9095 ^¶^**
3	4 (8%)	2 (7.7%)	2 (8.7%)	
4	21 (42%)	12 (46.2%)	9 (39.1%)	
≥5	24 (48%)	12 (46.2%)	12 (52.2%)	

Abbreviation: NAS, non-alcoholic steatohepatitis score. **^¶^** Fischer’s exact test.

**Table 2:** Magnetic resonance images features and NAFLD score classification of the patients with nonalcoholic steatohepatitis at the baseline.

Variable	Total (*n* = 50)	Study Group (*n* = 27)	Control Group (*n* = 23)	*p* Value
SteatosisGrades on MRI **				
MRI PDFF (median, range)	9 (2.9; 27.4)	14.9 (2.9; 27.4)	6.4 (3.9; 23.4)	0.040 ^†^
Grades 0–1 steatosis (*n*/%)	35 (70.0%)	16 (59.2%)	19 (82.6%)	0.073 *
Grades 2–3 steatosis (*n*/%)	15 (30.0%)	11 (40.7%)	4 (17.4%)	
**Fibrosis on Elastography**				
Shear stiffness (kPa) (median, range)	3.23 (2.31; 8.74)	3.41 (2.46; 7.59)	3.05(2.31; 8.74)	0.416 ^†^
Normal liver tissue (*n*/%)	16 (34.0%)	7 (29.2%)	9 (39.1%)	0.471 *
Any grade of fibrosis (*n*/%)	31 (66.0%)	17 (70.8%)	14 (60.9%)	
**NAFLD Fibrosis Score**				0.552 ^¶^
Absence of significant fibrosis (*n*/%)	17 (43.0%)	9 (33.3%)	8 (34.8%)	
Indeterminate (*n*/%)	25 (50.0%)	15 (55.6%)	10 (43.5%)	
Presence of significant fibrosis (*n*/%)	8 (16.0%)	3 (11.1%)	5 (21.7%)	

Abbreviations: MRI: magnetic resonance imaging; PDFF: hepatic proton density fat fraction; kPa: kiloPascal; NAFLD: nonalcoholic fatty liver disease; Classification of the grade of steatosis according MRI PDFF: ** grades 0–1 steatosis: PDFF <17.4%; grades 2–3 steatosis: PDFF 17.4%–22.1%; ^†^ Mann-Whitney U test; * Pearson Chi-square test; **^¶^** Fischer’s exact test.

**Table 3:** Baseline metabolic characteristics at enrollment of the patients with nonalcoholic steatohepatitis.

Variable	Total (*n* = 50)	Study Group (*n* = 27)	Control Group (*n* = 23)	*p* Value
**Metabolic Characteristics**				
Obesity (*n*/%)	49 (98.0%)	27 (100.0%)	22 (95.3%)	0.460 ^¶^
High body fat percentage (*n*/%)	40 (87.0%)	23 (85.0%)	17 (88.5%)	1.000 ^¶^
Hypertension (*n*/%)	38 (76.0%)	19 (70.4%)	19 (82.6%)	0.313 *
Hypercholesterolemia (*n*/%)	38 (76.0%)	21 (77.8%)	17 (73.9%)	0.750 *
Low HDL-c (*n*/%)	20 (40.0%)	11 (40.7%)	9 (39.1%)	0.980 *
Hypertriglyceridemia (*n*/%)	31 (62.0%)	19 (70.4%)	12 (52.2%)	0.186 *
Insulin resistance	7 (14%)	7 (25.0%)	0 (0%)	0.176 ^§^
Glucose intolerance (*n*/%)	14 (28.0%)	6 (21.4%)	8 (36.4%)	
Type 2 diabetes	19 (38.0%)	9 (32.1%)	10 (45.5%)	
Metabolic Syndrome (*n*/%)	49 (98.0%)	26 (96.3%)	23 (100.0%)	1.000 **^¶^**
Sedentarism	35 (70.0%)	16 (59.3%)	16 (82.6%)	0.073 *
**Hepatic Biochemistry**				
AST (× ref. value) (median, range)	0.9 (0.4;3.8)	0.9 (0.4;3.8)	0.9 (0.5;3.7)	0.869 ^†^
ALT (× ref. value) (median, range)	0.9 (0.3;5.5)	0.9 (0.3;5.5)	0.9 (0.4;3.2)	0.899 ^†^
ALP (× ref. value) (median, range)	0.7 (0.3;7.4)	0.7 (0.3;7.4)	0.7 (0.5;3.1)	0.442 ^†^
GGT (× ref. value) (median, range)	1.7 (0.3;21.7)	1.6 (0.5;17.1)	1.8 (0.3;21.7)	0.719 ^†^
Albumin (g/dL) (mean ± SD)	4.4 ± 0.3	4.4 ± 0.3	4.3 ± 0.4	0.429 ^#^
Total bilirubin (mg/dL) (median, range)	0.6 (0.2;2.4)	0.6 (0.2;2.4)	0.6 (0.3;2.3)	0.5000 ^†^
Platelets (/mm^3^) (mean ± SD)	239,560 ± 75,955	242,555 ± 71,910	236,043 ± 81,941	0.766 ^#^

Abbreviations: HDL: high density lipoprotein cholesterol; AST: aspartate aminotransferase; ALT: alanine aminotransferase; ALP: alkaline phosphatase; GGT: gamma-glutamiltransferase; Ref. value: reference value; SD: standard deviation. * Pearson Chi-square test; ^¶^ Fischer’s exact test; ^†^ Wilcoxon W test; ^#^ *t*-test; ^§^ Chi-square test for linear trend.

**Table 4:** Baseline clinical characteristics at enrollment of the patients with nonalcoholic steatohepatitis.

Intestinal Parameters	Total (*n* = 50)	Study Group (*n* = 27)	Control Group (*n* = 23)	*p* Value
SIBO (*n*/%)	2 (4.0%)	1 (3.7%)	1 (4.3%)	1.00 ^¶^
LPS (EU/mL) (median, range)	0.67 (0.28;1.66)	0.69 (0.34;1.43)	0.63 (0.28;1.66)	0.365 ^†^
**Intestinal Permeability Test**				
% lactulose excretion (median, range)	0.222 (0.010;1.140)	0.270 (0.010;0.590)	0.175 (0.010;1.140)	0.780 ^†^
Altered excretion of lactulose	23 (51.1%)	15 (55.6%)	8 (44.4%)	0.465 *
Normal excretion of lactulose	22 (48.9%)	12 (44.4%)	10 (55.6%)	
% mannitol excretion (mean ± SD)	17.65±6.61	16.61 ± 5.53	18.70 ± 7.69	0.294 ^#^
Altered excretion of mannitol	2 (4.4%)	1 (3.7%)	1 (5.6%)	1.00 ^¶^
Normal excretion of mannitol	43 (95.6%)	26 (96.3%)	17 (94.4%)	
Lactulose/mannitol (median, range)	0.014 (0.001;0.146)	0.016 (0.001;0.146)	0.011 (0.001;0.116)	0.677 ^†^
Altered ratio lactulose/mannitol	23 (51.1%)	15 (55.6%)	8 (44.4%)	0.465 *
Normal ratio lactulose/mannitol	22 (48.9%)	12 (44.4%)	10 (55.6%)	

Intestinal permeability test: study group, *n* = 27 and control group, *n* = 18; results are reported as percent of ingested sugar. Abbreviations: SIBO: small intestinal bacterial overgrowth; LPS: lipopolysaccharide. * Pearson Chi-square test; ^¶^ Fischer’s exact test; ^†^ Wilcoxon W test; ^#^ *t*-test.

**Table 5:** Comparison of the anthropometric and biochemical parameters before and after synbiotic supplementation.

Variable	Study Group			Control Group		
	Before	After	*p* Value	Before	After	*p* Value
**Metabolic Variables**						
Body weight (kg)	85.2 ± 14.6	83.9 ± 14.0	**0.006 ^#^**	84.5 ± 20.4	84.5 ± 20.2	0.837 ^†^
BMI (kg/m^2^)	32.5 ± 4.0	32.1 ± 3.8	**0.005 ^#^**	32.5 ± 4.0	32.3 ± 5.8	0.924 ^#^
WC (cm)	107.8 ± 10.8	105.9 ± 11.2	**0.001 ^#^**	104.0 ± 13.55	104.9 ±13.8	0.600 ^#^
Body fat (%) *	37.3 (27.2; 44.1)	36.6 (11.8; 45.0)	0.987 ^†^	32.6 ± 5.85	31.5 (22.1; 45.8)	0.576 ^#^
BEE (kcal) *	1645 (1232; 2599)	1545 (1207; 2508)	0.256 ^†^	1560 (11,061; 3191)	1609 (1345; 2980)	0.573 ^†^
Ferritin (ng/dL)	129 (23.3; 685.0)	131 (14.2; 530.0)	0.903 ^†^	134 (16.2; 377.5)	151 (11.1; 943)	0.920 ^†^
Glucose (mg/dL)	99.0 (80; 293)	101.0 (83; 322)	0.207 ^†^	109 (85; 280)	127 (84; 326)	0.123 ^†^
Cholesterol (mg/dL)	205.4 ± 34.6	208.9 ± 38.4	0.605 ^#^	190.9 ± 42.5	195.5 ± 30.4	0.458 ^#^
LDL-c (mg/dL)	125.2 ± 28.0	125.8 ± 35.5	0.928 ^#^	109.3 ± 32.7	104.2 ± 24.8	0.365 ^#^
HDL-c (mg/dL)	44.9 ± 10.3	44.3 ± 8.9	0.685 ^#^	46.7 ± 11.7	46.2 ± 10.5	0.822 ^#^
VLDL-c (mg/dL)	35 (20; 66)	35 (16; 122)	0.485 ^†^	29 (13.9; 130)	37 (13.3; 133)	**0.006 ^†^**
Triglycerides (mg/dL)	176 (100; 328)	173 (81; 572)	0.461 ^†^	147 (69–610)	183 (66.4; 663)	**0.006 ^†^**
Uric acid (mg/dL)	5.5 ± 1.4	4.9±1.2	**0.006 ^#^**	5.7 ± 1.9	5.2 ± 1.6	0.167 ^†^
**Liver Biochemistry and Platelet Count**						
ALT (× ref. value)	0.9 (0.3; 5.5)	0.8 (0.2; 4.5)	0.568 ^†^	0.9 (0.4; 3.1)	1.0 (0.4; 4.4)	0.738 ^†^
AST (× ref. value)	0.9 (0.4; 3.8)	0.9 (0.3; 4.1)	0.422 ^†^	0.9 (0.5; 3.7)	0.9 (0.3; 4.3)	0.584 ^†^
ALP (× ref. value)	0.7 (0.3; 7.4)	0.7 (0.3; 1.2)	0.939 ^†^	0.7 (0.5; 3.1)	0.8 (0.5; 2.5)	0.196 ^†^
GGT (× ref. value)	1.6 (0.5; 17.1)	1.7 (0.6; 9.4)	0.990 ^†^	1.8 (0.3; 21.7)	1.5 (0.5; 12.1)	0.858 ^†^
Albumin (mg/dL)	4.4 ± 0.3	4.4 ± 0.4	0.194 ^#^	4.3 ± 0.4	4.2 ± 0.4	0.173 ^#^
Total bilirubin (mg/dL)	0.6 (0.2; 2.4)	0.5 (0.3; 2.2)	0.137 ^†^	0.6 (0.3; 2.3)	0.7 (0.3; 2.3)	0.179 ^†^
Platelets (/mm^3^)	242,556 ± 71,911	239,519 ± 85,325	0.518 ^†^	236,044 ± 242,556	248,391 ± 78,480	0.078 ^#^

Abbreviations: BMI: body mass index; WC: waist circumference; BEE: basal energy expenditure (* estimated by biompedance); LDLc: low density lipoprotein cholesterol; HDL-c: high density lipoprotein; cholesterol, VLDL-c: very low density lipoprotein cholesterol; AST: aspartate aminotransferase, ALT: alanine aminotransferase; ALP: alkaline phosphatase; GGT: gamma-glutamiltransferase; ref. value: reference value. Variables expressed by means ± standard deviations: body weight, BMI, WC, cholesterol, LDL-c, HDL-c, uric acid, albumin and platelets. The other variables are expressed by the median values and range. * Pearson Chi-square test; ^†^ Wilcoxon W test; ^#^ *t*-test.

## Abbreviations

The following abbreviations are used in this manuscript:

NAFLD: nonalcoholic fatty liver disease; NASH: nonalcoholic steatohepatitis; T2DM: type 2 diabetes mellitus; IR: insulin resistance; SIBO: small intestine bacterial overgrowth; LPS: lipopolysaccharide; SCFAs: short chain fatty acids; CRN: Clinical Research Network; MRI: magnetic resonance imaging; ALT: alanine aminotransferase; AST: aspartate aminotransferase; GGT: gamma-glutamiltransferase; AP: alkaline phosphatase; HOMA: homeostasis model assessment; BMI: body mass index; MS: metabolic syndrome; PDFF: proton density fat fraction; MR: magnetic resonance; kPa: kiloPascal; HPLC: high performance liquid chromatography; H2BT: glucose hydrogen breath test; CFU: colony forming units; HDL-c: high density lipoprotein cholesterol; VLDL-c: very low density lipoprotein cholesterol; WC: waist circumference; BEE: basal energy expenditure; LDL-c: low density lipoprotein cholesterol; Cpt1a: carnitine palmitoyltransferase; GLP-1: glucagon-like pepetide-1; TRL4: toll like receptor 4; SREBP1-c: sterol regulator element-binding protein-1 c; IL: interleukin.
